# Dementia in type 2 diabetes: risk factors, disease processes and opportunities for prevention across the lifespan

**DOI:** 10.1007/s00125-026-06795-2

**Published:** 2026-07-13

**Authors:** Jan F. de Leijer, Geert Jan Biessels, Frank L. J. Visseren, Thomas T. van Sloten

**Affiliations:** 1 Department of Vascular Medicine & Endocrinology, University Medical Center Utrecht, Utrecht, the Netherlands; 2 Department of Neurology, UMC Utrecht Brain Center, University Medical Center Utrecht, Utrecht, the Netherlands

**Keywords:** Dementia, Prevention, Probabilistic model, Review, Risk factors, Type 2 diabetes

## Abstract

People with type 2 diabetes have a 60–70% higher risk of developing dementia than those without diabetes. Given the increasing number of older people living with diabetes, preventing dementia in this population is becoming increasingly important. However, dementia in individuals with type 2 diabetes is not a single entity and no diabetes-specific pathologies contributing to dementia have been identified yet. Here, we suggest that the relation between type 2 diabetes and dementia may be best understood through a probabilistic model that considers the context in which type 2 diabetes develops and the involvement of multiple dementia-related pathologies. Within this model, type 2 diabetes alters both the probability of dementia-related pathologies occurring and their downstream consequences. Opportunities for prevention of dementia evolve across the lifespan and comprise reducing cumulative exposures to dementia risk factors through a holistic approach rather than through targeting a single factor. Accordingly, beyond preventing type 2 diabetes itself, glucose-lowering medications with effects beyond glycaemic management (e.g. multihormone receptor modulators, such as glucagon-like peptide-1 receptor agonists, and sodium–glucose cotransporter 2 inhibitors), alongside traditional multi-domain cardiovascular risk management, may be most effective at lowering excess dementia risk in individuals with type 2 diabetes.

**Graphical Abstract:**

**Figure:**
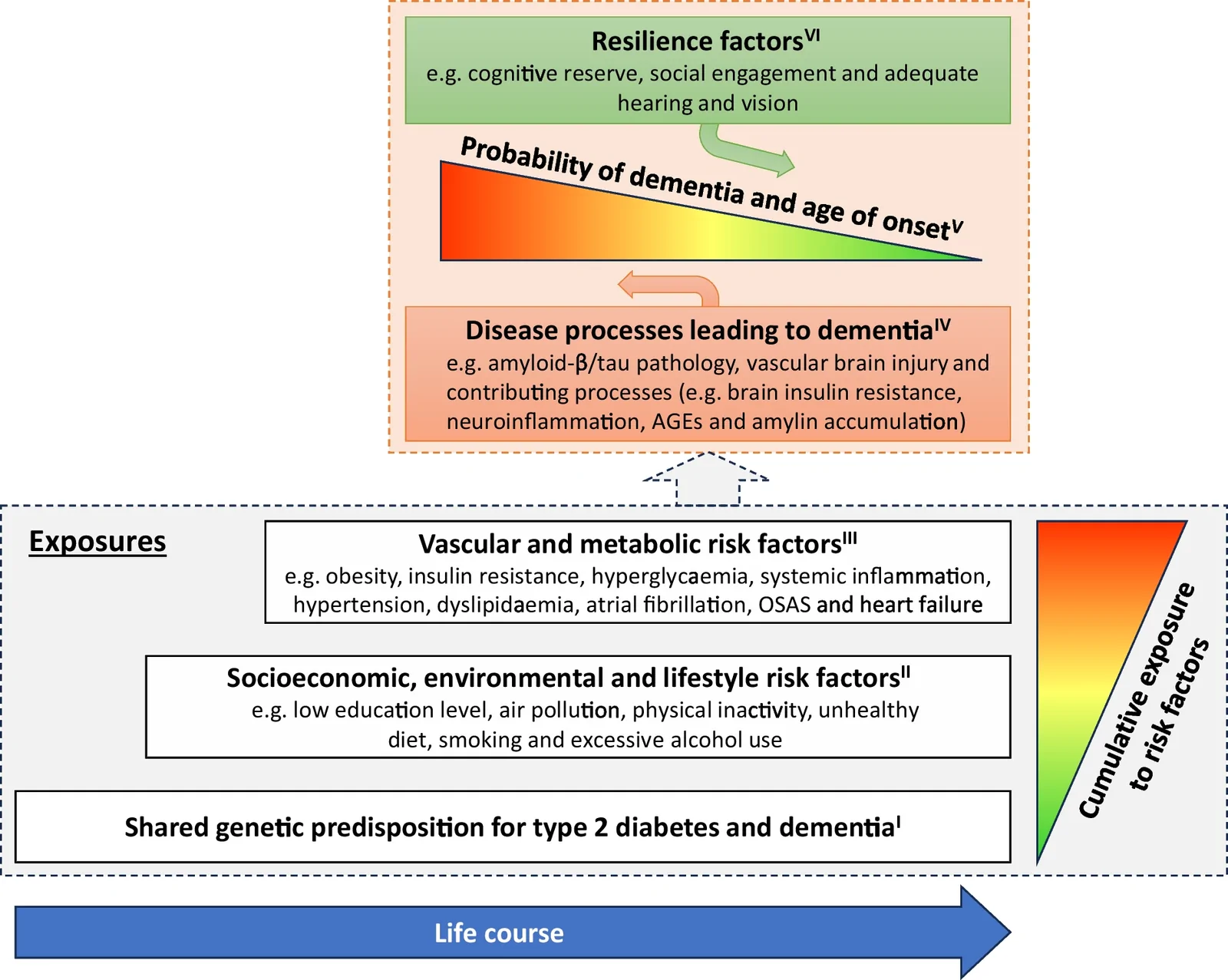

**Supplementary Information:**

The online version contains a slideset of the figures for download available at https://doi.org/10.1007/s00125-026-06795-2.

## Introduction

Worldwide, nearly one in four people aged 65–99 years (158 million) are living with diabetes [1]. This number is projected to reach 278 million by 2050 [1]. Over the past two decades, therapeutic progress in reducing cardiovascular disease and mortality in people with type 2 diabetes has contributed to longer life expectancy. As a result, older people (aged >65 years) now make up a substantial and growing proportion of those with diabetes and face health complications associated with both diabetes and ageing. Notably, this includes dementia. Observational studies consistently show that individuals with type 2 diabetes have an approximately 60–70% higher risk of developing all-cause dementia than those without type 2 diabetes, with a comparable excess risk in men and women [2]. For example, in a population-based study from France, the estimated 12 year absolute risk of dementia for a 70-year-old man with a low educational level was 23% among those with type 2 diabetes and 14% among those without diabetes [3]. Dementia risk among individuals with type 2 diabetes may also differ across ethnic groups, with higher risk observed in African American and Native American individuals than in Asian American and White individuals [4, 5]. However, it remains unclear to what extent these differences reflect inherent biological, particularly genetic, variation rather than differences in socioeconomic, environmental and lifestyle factors. Dementia has a profound negative impact on the quality of life of those affected and their families. It also impairs an individual’s ability to manage diabetes effectively and is associated with a higher risk of glycaemic emergencies [6]. Prevention of dementia in individuals with type 2 diabetes is therefore critically important.

Of note, dementia is not a single entity, but a syndrome, resulting from multiple pathologies, of which Alzheimer-type and vascular pathologies are the most common. Over the past decades, these pathologies have largely been approached as distinct entities, with different diagnostic criteria aiming to distinguish one from the other. In reality, the vast majority of people who develop dementia at an older age have mixed pathologies, even if one pathology may be dominant [7]. This does not mean to say that all dementia is a ‘mixed bag’. Specific pathologies result from specific disease processes that may provide leads for therapy. Most recent efforts to devise such therapies have focused on Alzheimer’s disease, driven by the amyloid hypothesis. At the core of this lies the long-held belief that a deterministic chain of events leads from amyloid and tau deposition to neurodegeneration and subsequent dementia [8]. Fed by observations from trials in which amyloid-lowering therapies managed to reduce amyloid burden, but much less so cognitive decline, other views are emerging. Particularly relevant for diabetes, a probabilistic model of Alzheimer’s disease was recently proposed (Text box 1) that likely applies equally to other dementia types [8]. This model postulates that, particularly in sporadic Alzheimer’s disease, the downstream consequences of the amyloid pathophysiological cascade are to a substantial extent determined by a range of stochastic factors (e.g. environmental exposures and polygenic risk), accounting for neuropathological and clinical variability [8].

**Fig. 1:**
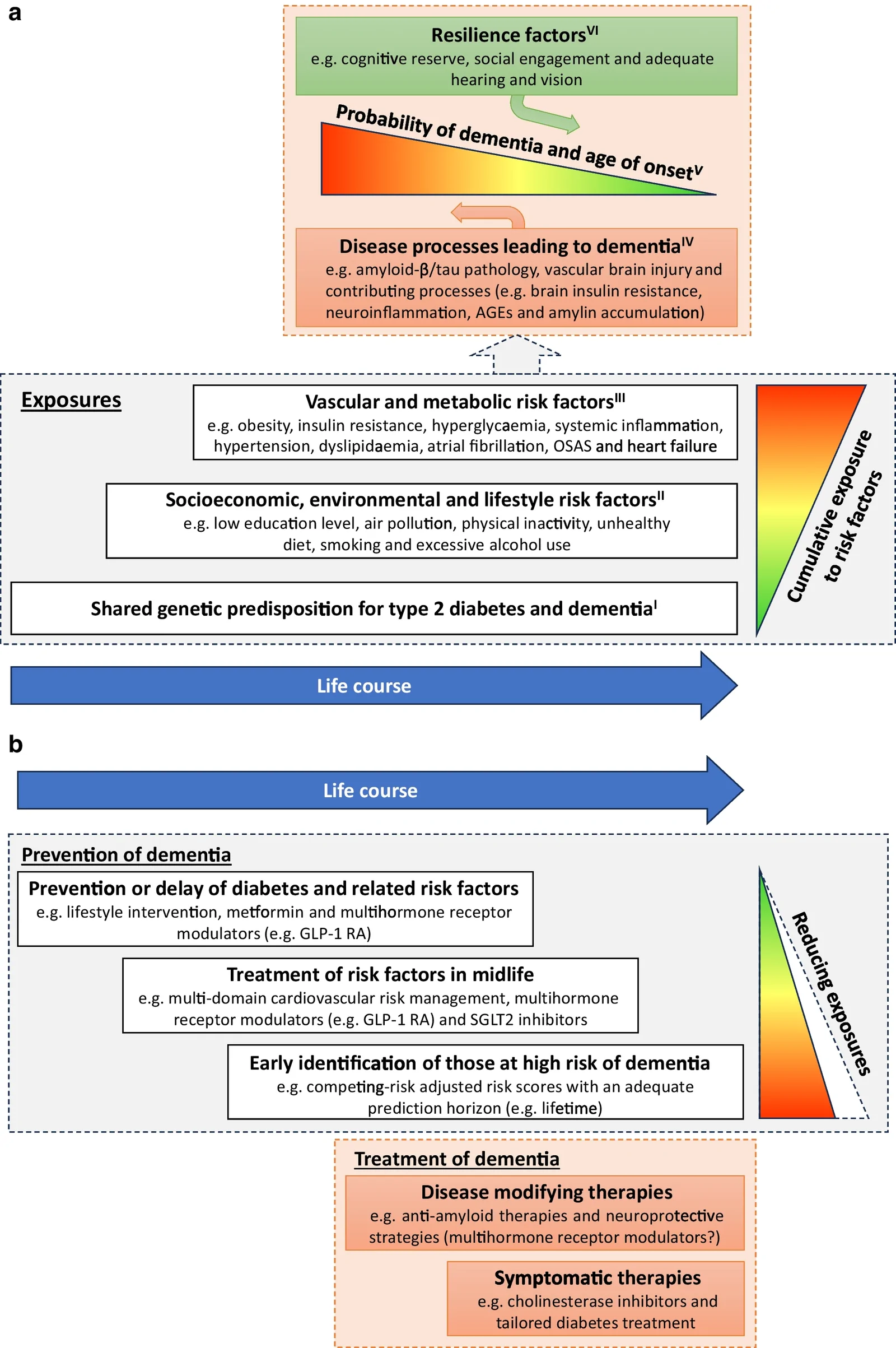
(**a**) Proposed probabilistic framework for all-cause dementia in relation to type 2 diabetes across the lifespan. Type 2 diabetes-associated dementia risk is partially attributable to a substantial overlap in genetic (I) and acquired (II) predisposition for the two conditions. When type 2 diabetes occurs, it often does so in the context of a broad spectrum of vascular and metabolic risk factors (III) for dementia. The higher the cumulative exposure to these risk factors, the greater their impact on the disease processes that lead to dementia (IV). Dementia occurs only when key dementia pathologies are present (i.e. amyloid-β/tau pathology and vascular brain injury) but not everyone who has these pathologies goes on to develop dementia. The probability of dementia occurrence and age at onset (V) depends on a wide range of stochastic factors (e.g. contributing disease processes and resilience factors [VI]), many of which are influenced by type 2 diabetes. (**b**) Proposed windows of opportunity for prevention and treatment of dementia in the context of type 2 diabetes. Before onset of dementia, efforts should be directed at reducing cumulative exposure to dementia risk factors. After onset of dementia, selected individuals may benefit from disease-modifying and symptomatic therapies to slow clinical progression and alleviate symptoms. GLP-1 RA, GLP-1 receptor agonist; OSAS, obstructive sleep apnoea syndrome. This figure is available as a downloadable slideset

In our view, the relation between type 2 diabetes and dementia is best understood through such a probabilistic model, considering mixed pathologies and taking a lifespan perspective. We will apply the probabilistic model to all-cause dementia in relation to diabetes, considering how type 2 diabetes and dementia may in part emerge through shared risk factors and how, once diabetes develops, diabetes-related factors may aggravate dementia risk (Fig. 1a; definitions in Text box 1). We also discuss how this probabilistic model may inform on optimal windows of opportunity for preventing and treating dementia in relation to diabetes across the lifespan (Fig. 1b).

**Figure:**
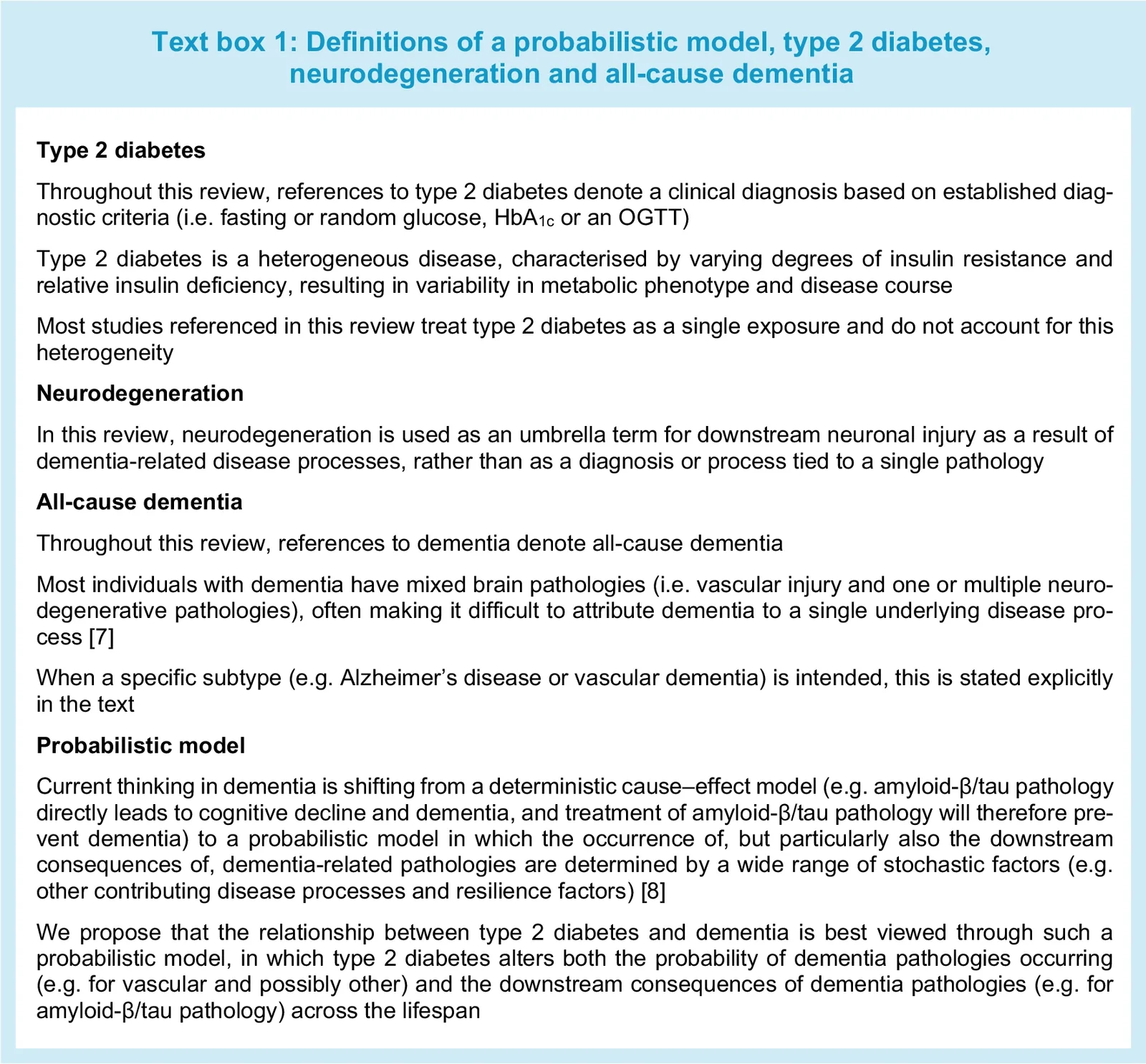

## Prediabetic stages: shared predisposition for type 2 diabetes and dementia

Various genetic and acquired factors predispose people to both type 2 diabetes and dementia. Those who actually acquire type 2 diabetes may be at even higher dementia risk, through exposure to diabetes-related metabolic disturbances and complications.

### Genetic predisposition

The risk of both type 2 diabetes and dementia is dependent on the interplay of multiple genetic factors. Although monogenic forms of diabetes (e.g. MODY) and dementia (e.g. autosomal dominant Alzheimer’s disease) exist, they account for less than 5% of diabetes and 1% of dementia cases, respectively [8]. Overall, genetic factors are estimated to account for 40–70% of the variance in liability for type 2 diabetes and dementia [9, 10]. Importantly, some of the genetic factors underlying type 2 diabetes and dementia overlap [11, 12]. One study comparing genome-wide association studies identified nearly 400 SNPs associated with both type 2 diabetes and Alzheimer’s disease, each with small individual effects [12]. For instance, the APOE*E4 allele, the primary genetic risk factor for Alzheimer’s disease, has also been associated with an 18% increase in the odds of type 2 diabetes [13]. In line with this, another study found that type 2 diabetes-associated genetic variants were associated with a higher risk of all-cause dementia [11]. The observed genetic overlap between diabetes and dementia suggests that these conditions may share biological pathways that contribute to both metabolic dysfunction and neurodegenerative processes across the lifespan.

Genetic susceptibility may also modify the association between type 2 diabetes and dementia risk. The most studied interaction involves *APOE* genotype but findings are mixed regarding direction and magnitude. Some studies [14, 15] report that type 2 diabetes confers a greater relative increase in dementia risk among APOE*E4 carriers (suggesting synergistic effects), whereas others [16, 17] report a smaller incremental diabetes-associated risk among APOE*E4 carriers (suggesting effect attenuation).

### Gestational diabetes mellitus and birthweight

Gestational diabetes mellitus might be a shared risk factor for type 2 diabetes and dementia in both mother and offspring. In a study using data from over 200,000 women from the UK Biobank, those with a history of gestational diabetes mellitus had a 67% higher risk of dementia than those without, of which 35% was mediated by incident type 2 diabetes [18]. No studies have examined whether gestational diabetes mellitus affects dementia risk in the offspring in later life. However, a growing body of evidence shows that gestational diabetes mellitus is associated with adverse neurodevelopmental outcomes in children, possibly predisposing to accelerated brain ageing later in life [19]. In line with this, birthweight has been associated with later-life risk of both type 2 diabetes and dementia in a J-shaped pattern, such that individuals born with low or high birthweight might have an increased risk of both diabetes and dementia compared with those with mid-range birthweights [20, 21].

### Socioeconomic, environmental and lifestyle factors

After birth, various socioeconomic (e.g. education level), environmental (e.g. air pollution) and lifestyle (e.g. physical inactivity, unhealthy diet, smoking and excessive alcohol consumption) factors further predispose to developing both type 2 diabetes and dementia [22, 23]. Although the relative influence of these exposures may differ between the two conditions, a strong overlap is evident. For example, smoking has been shown to increase the risk of type 2 diabetes by 44% and the risk of dementia by 30% [24, 25]. Similarly, a 10 μg/m^3^ increase in fine particulate matter (PM_2.5_) air pollution has been linked to an 11% higher risk of type 2 diabetes and a 34% higher risk of dementia [26, 27]. In line with the probabilistic framework for dementia, exposome data suggest that combined socioeconomic, environmental and lifestyle factors may shape brain ageing more than isolated exposures [28].

## Risk factors for dementia across prediabetic and diabetic stages

### Hyperglycaemia and insulin resistance

In individuals with type 2 diabetes, but also in prediabetic stages, higher HbA_1c_ and fasting plasma glucose levels and higher insulin resistance have been associated with a greater dementia risk in observational studies [29–33]. For instance, individuals with type 2 diabetes and HbA_1c_ levels above 75 mmol/mol (9%) have a 31–93% higher risk of dementia than individuals with type 2 diabetes and lower HbA_1c_ levels [29, 30]. Similarly, individuals with prediabetes (i.e. HbA_1c_ 39–46 mmol/mol [5.7–6.4%]) have a 12% higher risk of dementia than those without prediabetes (i.e. HbA_1c_ <39 mmol/mol [<5.7%]) [32]. Individuals with type 2 diabetes with the greatest between-measurement HbA_1c_ variability also have a 40–46% higher risk of dementia [34]. However, these relative risks derived from observational studies should be interpreted with caution, given that they are prone to confounding and reverse causation. The latter is especially important in this context, because poor cognitive function increases the risk of chronic hyperglycaemia [6].

In the past decade, Mendelian randomisation studies have been used to assess whether the observed associations between type 2 diabetes, glycaemic traits (i.e. hyperglycaemia and insulin resistance) and dementia are of causal nature. Supporting a causal link, most Mendelian randomisation studies have found an association between genetically predicted type 2 diabetes and vascular dementia and all-cause dementia [17, 35, 36]. In line with this, one study has also found an association between genetically predicted high plasma glucose levels and the risk of all-cause dementia [37]. In contrast, most Mendelian randomisation studies have found no association between genetically predicted type 2 diabetes and Alzheimer’s disease [38]. Similarly, no consistent relation between genetically predicted higher plasma glucose levels, higher HbA_1c_ levels or more severe insulin resistance and Alzheimer’s disease risk has been found [37, 39–43]. This pattern suggests that diabetes may contribute more strongly to vascular and mixed dementia pathways than to primary Alzheimer’s disease pathology.

### Comorbidities of type 2 diabetes

Type 2 diabetes often develops in the context of various adverse conditions, including obesity, low-grade systemic inflammation, hypertension, atrial fibrillation, obstructive sleep apnoea syndrome, heart failure and dyslipidaemia [44–47]. Each of these conditions may contribute to a higher dementia risk. For instance, meta-analyses of observational studies show that individuals with midlife hypertension have a 20% higher risk of dementia and those with low-grade inflammation have a 37% higher risk [48, 49].

Multiple studies have shown that the effect of type 2 diabetes on dementia risk is strongly dependent on the age at onset of type 2 diabetes; an earlier onset confers a higher relative risk of dementia compared with an onset at older age, as well as a higher absolute lifetime risk [50, 51]. For example, a study using prospective data from the Whitehall II study found that compared with a type 2 diabetes onset at 70 years, every 5 year earlier onset of type 2 diabetes was associated with a 24% higher relative risk of dementia [51]. Similarly, a study using data from the ARIC (atherosclerosis risk in communities) study showed that the competing risk-adjusted lifetime risk of dementia was higher among individuals with middle age-onset type 2 diabetes than among those with older-onset type 2 diabetes (36% and 31%, respectively) [50]. These associations may be partly explained by longer exposure to type 2 diabetes. From a lifespan perspective, however, the excess dementia risk in early-onset type 2 diabetes may also reflect that these individuals represent a subgroup with a higher predisposition to type 2 diabetes and thus possibly also to dementia. For example, the higher levels of obesity or ectopic fat often required to develop type 2 diabetes at a younger age are accompanied by more adverse lipid profiles, higher BP and faster glycaemic deterioration compared with later-onset type 2 diabetes [44]. All these factors may also increase the risk of dementia.

## Dementia-related pathologies and diabetes

Dementia in individuals with type 2 diabetes is not a single entity. Like in people without diabetes, it can be caused by multiple disease processes, often occurring in combination, including amyloid-β/tau pathology, macrovascular injury, small vessel injury and (downstream) neurodegeneration, the latter also related to other processes. At present, no diabetes-specific pathologies contributing to dementia have been identified. Dementia occurs only when key dementia pathologies are present (i.e. amyloid-β/tau pathology and vascular brain injury) but not everyone who has these pathologies develops dementia. Apart from the combined burden of these pathologies, the probability of dementia occurrence and age at onset depend on a wide range of stochastic factors (e.g. contributing disease processes and resilience factors). Below, we describe how type 2 diabetes may influence different dementia-related pathologies and resilience factors.

### Amyloid-β and tau pathology

Although observational studies show that type 2 diabetes increases the risk of a clinical diagnosis of Alzheimer’s disease [52], the burden of Alzheimer’s disease neuropathology (i.e. amyloid-β and tau deposits) does not appear to be increased in individuals with type 2 diabetes [53–56]. Specifically, type 2 diabetes in midlife is associated with greater tau pathology later in life but not with amyloid-β pathology as measured through positron emission tomography (PET) [53, 56]. In line with this, post-mortem neuropathological studies found that the amount of amyloid-β deposition is not higher in people with type 2 diabetes compared with those without diabetes [55]. The current framework for Alzheimer’s disease is that amyloid-β pathology emerges early and accumulates over a prolonged preclinical phase, while tau pathology develops subsequently and aligns more closely with neurodegeneration and the onset and progression of cognitive symptoms. If type 2 diabetes were to accelerate the development of Alzheimer’s disease pathologies, amyloid-β would be expected to aggregate before tau in the presence of type 2 diabetes [57]. Therefore, excess neurodegeneration in type 2 diabetes is likely due to pathways different from Alzheimer’s disease. However, type 2 diabetes may still increase the probability of a clinical Alzheimer’s diagnosis by lowering the threshold at which Alzheimer’s pathology becomes clinically apparent, and Alzheimer’s disease remains the most common dementia diagnosis among individuals with type 2 diabetes. In addition, diabetes-related decline in brain glucose metabolism could shift amyloid-β from a protective to a toxic form, without necessarily increasing its overall presence [58].

### Macrovascular injury

Excess dementia risk in type 2 diabetes is likely driven to some extent by vascular pathways [59, 60]. Type 2 diabetes accelerates atherosclerosis and stiffening of large arteries. A systematic review of 64 cohorts encompassing 775,000 individuals found that type 2 diabetes increases the risk of any stroke by 83–128% [61]. The risk of post-stroke dementia is approximately 1.6 times higher in individuals with type 2 diabetes than in those without [62]. In another study among post-stroke patients, the incidence of all-cause dementia in individuals with type 2 diabetes was 9% higher (19% vs 10%) than in individuals without type 2 diabetes [63].

### Small vessel injury

Type 2 diabetes is also associated with multiple features of cerebral small vessel disease [59, 60, 64, 65], which is the main vascular cause of dementia and may contribute to 45% of dementia cases [66, 67]. Observed structural features of cerebral small vessel disease on brain MRI in type 2 diabetes are described in Text box 2. Cerebral microvascular dysfunction, among others, is thought to underlie these structural features [59, 67, 68]. Evidence from neuropathology, neuroimaging and cerebrospinal fluid studies shows that type 2 diabetes-related cerebral microvascular dysfunction may encompass an increased blood–brain barrier permeability, perfusion defects and impaired neurovascular coupling and autoregulation, all of which may lead to neuronal injury and increase the risk of dementia [59].

**Figure:**
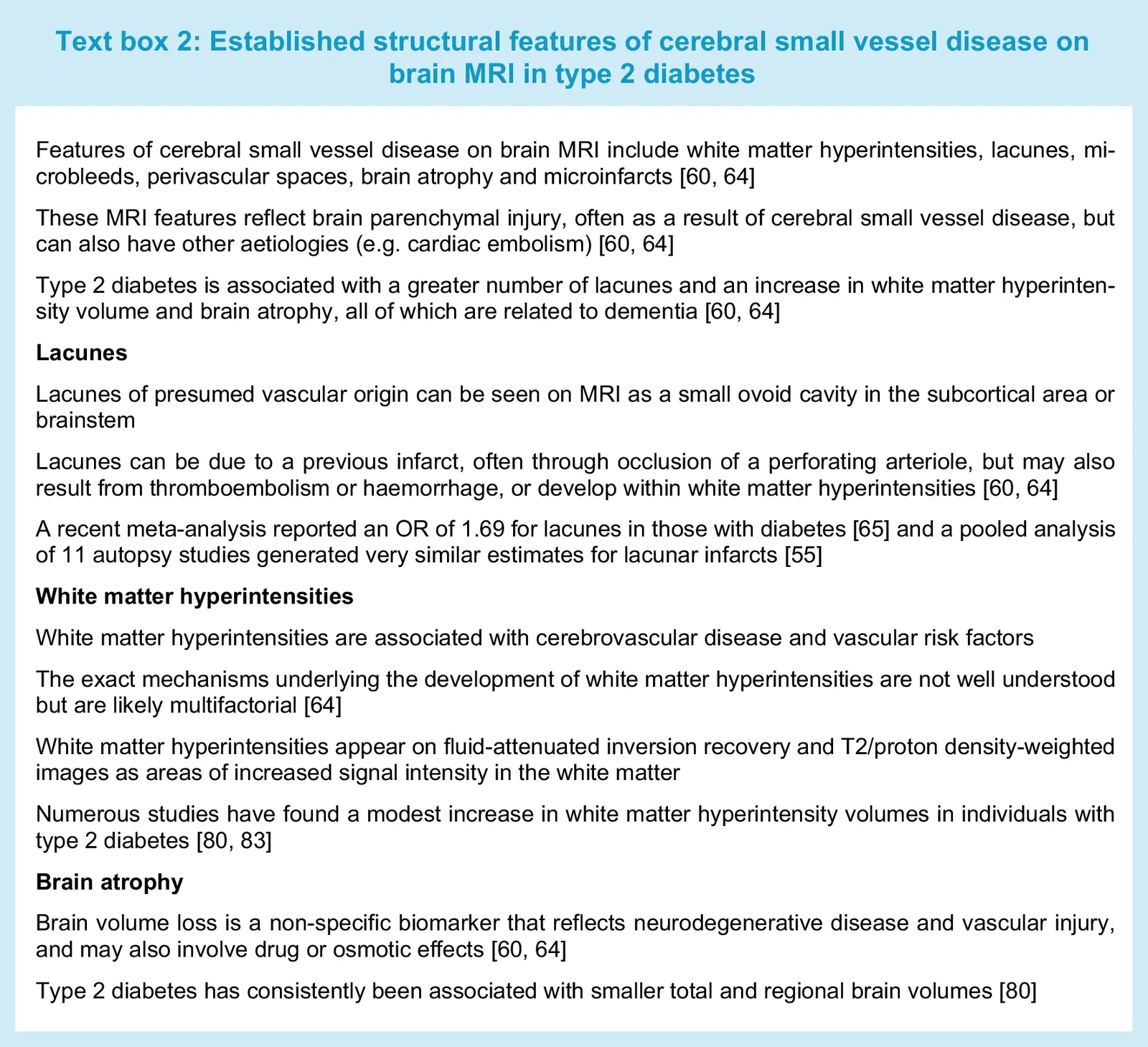

### Neurodegeneration related to other processes

Other processes that may contribute to neurodegeneration in type 2 diabetes include AGEs, brain insulin resistance, neuroinflammation and amylin accumulation (see Text box 3 for an explanation of these processes) [69–74]. These disease processes may enhance the development of key dementia pathologies (i.e. amyloid-β/tau pathology and vascular brain injury) but may also lower the threshold at which these pathologies translate into clinically apparent dementia.

**Figure:**
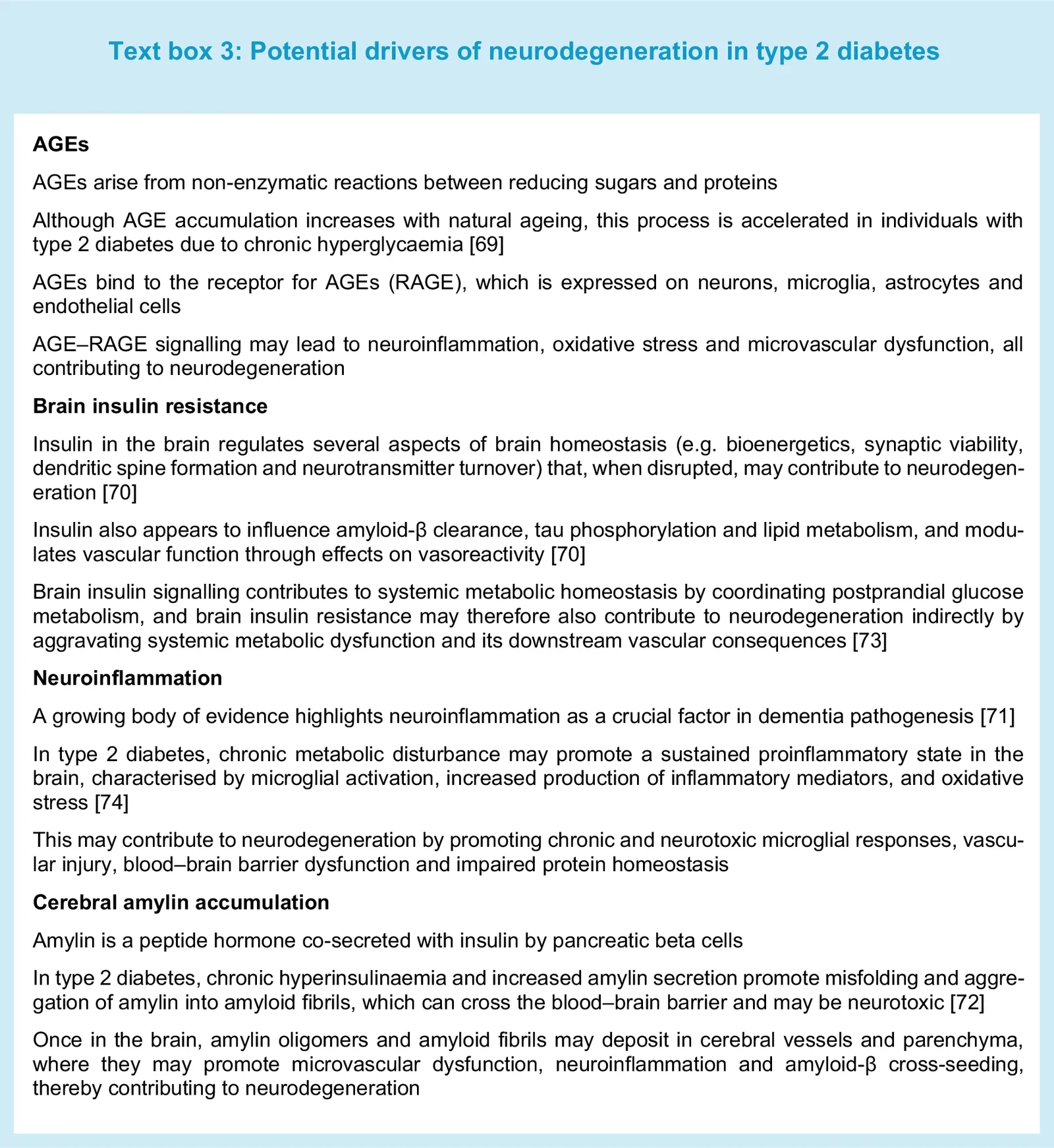

### Resilience factors

The probability and age of onset of dementia depend also on the presence of resilience factors. Type 2 diabetes is associated with a less-favourable dementia resilience profile. For example, individuals with type 2 diabetes have lower cognitive reserve, are more often socially isolated and are more likely to have hearing and vision loss compared with people without diabetes [75–77]. Consequently, individuals with type 2 diabetes may require a lower burden of dementia pathology before dementia becomes clinically apparent.

## Opportunities for prevention and treatment of dementia across the lifespan

Strategies for prevention and treatment of dementia in individuals with type 2 diabetes are strongly dependent on age. Early in life and in midlife, the greatest impact may come from broadly applicable strategies that lower cumulative exposure to modifiable risk factors (e.g. multi-domain cardiovascular risk management). In later life, focus may shift to identification of individuals at high risk of dementia and therapies that modify specific disease processes in selected individuals (e.g. anti-amyloid therapies).

### Prevention of type 2 diabetes and related vascular and metabolic risk factors

The incidence of young-onset type 2 diabetes is increasing worldwide [44, 78]. The first window of opportunity to reduce lifetime risk of dementia is therefore to prevent or delay type 2 diabetes onset (e.g. through lifestyle interventions, metformin and multihormone receptor modulators such as glucagon-like peptide-1 [GLP-1] receptor agonists) [79].

### Prevention of dementia with multi-domain cardiovascular risk management in midlife

Given that type 2 diabetes is often accompanied by a broad spectrum of vascular and metabolic risk factors for dementia, multi-domain cardiovascular risk management may be most effective in reducing dementia risk. However, a randomised clinical trial investigating the effect of multi-domain risk factor modification on dementia risk in individuals with type 2 diabetes is unlikely to be initiated. In addition to financial constraints, such a trial would raise ethical concerns because it would involve testing evidence-based care that is already recommended and known to reduce other adverse, particularly cardiovascular, outcomes. Therefore, in the absence of trial-level evidence for prevention strategies, evidence for earlier-life interventions depends on trials with intermediate outcomes (e.g. cognitive decline, imaging markers) and genetic or observational studies. Some of these studies supporting stringent management of cardiovascular risk factors to reduce the risk of dementia exist. An observational study using data from the UK Biobank (*n*=87,856) showed that the excess risk of dementia in individuals with type 2 diabetes was progressively lower in those who had a higher number of cardiovascular risk factors within target range, when compared with control individuals without type 2 diabetes [80]. Those with type 2 diabetes with at least five out of seven risk factors on target no longer had a statistically significant increased risk of developing dementia compared with control individuals (HR 1.32 [95% CI 0.89, 1.95]). Similarly, a healthy lifestyle and a low burden of cardiovascular risk factors have been shown to attenuate the accelerated brain ageing associated with type 2 diabetes [81]. The Look Action for Health in Type 2 diabetes (Look AHEAD) trial, a randomised clinical trial of 5145 individuals with type 2 diabetes, showed that a lifestyle intervention focused only on weight loss and physical exercise had a modest favourable effect on white matter hyperintensities and brain atrophy but no effect on dementia incidence [82, 83]. Other randomised clinical trials in individuals with and without diabetes in which multi-domain interventions were aimed at lifestyle factors (e.g. diet, physical activity, weight loss, smoking and alcohol use) and cardiovascular risk factors (e.g. BP and LDL-cholesterol) generally showed a small benefit on cognitive decline [84–86].

### Glucose-lowering therapies and dementia prevention

Trials investigating the effects of intensive glucose lowering on cognitive decline in individuals with type 2 diabetes, often as secondary outcome measure, have yielded largely negative results [87]. A key limitation of these studies is that they used cognitive decline, but not late-life dementia incidence, as the outcome. Diabetes-associated cognitive decrements are modest, progress slowly and are unlikely to indicate the earliest stage of dementia before the age of 65–70 years [88]. Therefore, due to the relatively young age of these trial populations at baseline (e.g. mean age 55–70 years at baseline) and short follow-up duration (e.g. median of 1–5 years), the a priori likelihood of detecting a treatment benefit was small. Intensive glucose lowering may also increase the risk of iatrogenic severe hypoglycaemic episodes, which have been associated with a higher risk of dementia in later life [89]. In a population-based study from Canada, a severe hypoglycaemic episode in midlife was linked to a threefold increase in the risk of dementia [89]. Additionally, intensive glycaemic management was achieved in these trials using medications with minimal effects beyond glucose lowering (e.g. sulfonylureas and insulin) that may not meaningfully influence the entire spectrum of adverse cardiometabolic changes in type 2 diabetes.

More recently, attention has shifted towards the potential cognitive benefits of newer glucose-lowering therapies of which the mechanism of action extends beyond glycaemic management. Evidence from observational studies suggests that both GLP-1 receptor agonists and sodium–glucose cotransporter 2 (SGLT2) inhibitors may reduce dementia risk, independent of their glucose-lowering effects [90–93]. In line with this, a Mendelian randomisation study showed that genetic variants linked to increased GLP-1 signalling, used as a proxy for a GLP-1 receptor agonist, were associated with a lower risk of Alzheimer’s disease [94]. Similarly, an exploratory analysis of the REWIND trial reported that treatment with the GLP-1 receptor agonist dulaglutide reduced the risk of substantive cognitive impairment by 14% over 5 years [95]. Substantive cognitive impairment was defined as a ≥1.5 SD decline in country-standardised Montreal Cognitive Assessment (MoCA) or Digit Symbol Substitution Test (DSST) scores. Furthermore, a recent systematic review and meta-analysis of randomised clinical trials, including participants with and without type 2 diabetes, found that GLP-1 receptor agonist use was associated with a 45% lower odds of dementia [96]. In these trials, dementia was recorded either as an adverse event or identified through ICD-10 codes. The same review found no significant effect of SGLT2 inhibitors on dementia risk, although this analysis was underpowered as both treatment arms included only 20–25 dementia events. An explanation for the potential cognitive benefits of multihormone receptor modulators and SGLT2 inhibitors is that their mechanisms of action extend beyond glucose lowering, encompassing anti-inflammatory, vascular and neuroprotective effects and that they may reduce brain insulin resistance. These findings, however, need to be confirmed by randomised clinical trials with dementia as a prespecified (secondary) outcome. The obvious question is whether such a trial is feasible given the long follow-up period needed to observe cases of incident dementia. An event-enriched trial design with older, high-risk participants and more pragmatic long-term follow-up may be required.

### Early identification of individuals at elevated risk of dementia

In older adults, priority may shift towards identifying those at highest risk of dementia to enable targeted screening and early detection. Early detection in these individuals is crucial, as diabetes treatment needs to be tailored to prevent chronic hyperglycaemia as well as severe hypoglycaemic episodes [97]. Several diabetes guidelines (e.g. ADA standards of care) recommend annual cognitive screening for early detection of mild cognitive impairment or dementia in all adults with type 2 diabetes aged 65 years or older [97]. However, implementation is difficult, in part because the high prevalence of type 2 diabetes in this age group strains clinical capacity [98]. To ensure adequate implementation, tailored strategies are necessary to determine who should undergo screening. A few studies have aimed to develop a risk score for predicting dementia risk in individuals with type 2 diabetes [99–101]. However, these models did not adjust for competing risks, were not externally validated in other cohorts, or included predictors that are not routinely measured in daily care. Furthermore, the use of these models is hampered by their limited prediction horizons, with a maximum of 10 years. Aside from determining who to screen, future research should determine the most appropriate screening tests and the optimal screening frequency [98].

### Disease-modifying drugs in late life

To date, no intervention has been shown to directly modify the disease processes leading to dementia in individuals with type 2 diabetes. Two recent repurposing trials of GLP-1 receptor agonists in individuals with early Alzheimer’s disease, but without type 2 diabetes, have been negative on their primary disease-modifying endpoints. In the phase 2 ELAD trial, the GLP-1 receptor agonist liraglutide did not significantly improve the primary outcome of cerebral glucose metabolism. However, for the secondary outcome (score on the Alzheimer’s Disease Assessment Scale-Executive domain [ADAS-Exec]), liraglutide performed better than placebo [102]. In the phase 3 EVOKE and EVOKE+ trials, orally administered semaglutide, a GLP-1 receptor agonist, did not demonstrate a statistically significant slowing of clinical progression vs placebo on the primary endpoint of clinical dementia rating [103]. However, it remains unclear whether the findings would be similar in individuals with diabetes. Furthermore, GLP-1 receptor agonists may be more effective in preventing dementia (as described in the previous paragraph) than treating it. Therapy with anti-amyloid monoclonal antibodies has been shown to lead to a modest decrease in cognitive decline in individuals with Alzheimer’s disease but without diabetes. However, targeting a single pathology such as amyloid may not be sufficient to truly modulate the type 2 diabetes-attributable burden of dementia.

## Conclusion

Prevention of excess dementia risk in type 2 diabetes requires a holistic approach that integrates the context in which type 2 diabetes develops. Here we described how risk factors predisposing to both dementia and type 2 diabetes, and later on in the process, diabetes-related factors, form a probabilistic web of interrelated risk factors that together shape an individual’s risk of dementia throughout the lifespan. Across individuals, this path can involve different dementia-related pathologies, often in combination. Accordingly, targeting single factors may benefit specific subgroups of individuals, although currently there is not a single candidate factor that can be targeted to substantially reduce the overall diabetes-associated burden of dementia.

Opportunities for prevention and treatment of dementia evolve across the lifespan. Current evidence suggests that multihormone receptor modulators and SGLT2 inhibitors in addition to stringent cardiovascular risk management in midlife may be most effective at lowering dementia risk in individuals with established type 2 diabetes. However, future trials with dementia as a prespecified outcome need to verify this. A challenge in determining optimal prevention strategies throughout the life course is that the mechanisms underlying excess neurodegeneration in type 2 diabetes remain incompletely understood [104]. As this is likely not caused by an increase in Alzheimer’s disease pathology, mechanistic studies of other neurodegeneration pathways (e.g. microvascular and neuroinflammatory) may identify new targets for prevention.

## Supplementary Information

Below is the link to the electronic supplementary material.

Slideset of figures (PPTX 280 KB)

## Abbreviations

GLP-1: Glucagon-like peptide-1
RAGE: Receptor for AGEs
SGLT2: Sodium–glucose cotransporter 2
